# Foreign Body Ingestion: A Common Presentation Among Pediatric Age Group in the City of AlAhsa Eastern Province, Saudi Arabia

**DOI:** 10.7759/cureus.31494

**Published:** 2022-11-14

**Authors:** Hussain A Al Ghadeer, Sajjad M AlKadhem, Alla M Albisher, Nouh H AlAli, Ali Salman Al Hassan, Murtadha H Alrashed, Mohammed H Alali, Raghad T Alturaifi, Mohammed B Alabdullah, Ahmed H Buzaid, Zakariya A Aldandan, Mohammed H Alnasser, Nasser S Aldandan, Abdulmohsen A Aljaziri

**Affiliations:** 1 Pediatrics, Maternity and Children Hospital, AlAhsa, SAU; 2 Pediatric Emergency Medicine, Maternity and Children Hospital, AlAhsa, SAU; 3 Pediatric Gastroenterology, Maternity and Children Hospital, AlAhsa, SAU; 4 Pediatrics, King Faisal University, AlAhsa, SAU

**Keywords:** alahsa., saudi arabia, ingestion, foreign body, endoscopy

## Abstract

Background

Foreign body ingestion is a prevalent issue among children and presents considerable morbidity and mortality rates. Due to children’s increased accessibility to electronic toys and equipment, foreign body ingestion has become a common reason for presenting to pediatric emergency departments worldwide. In this context, this research aims to determine the prevalence of foreign body ingestion among children in AlAhsa, Saudi Arabia.

Methodology

This observational retrospective descriptive study was conducted at Maternity and Children Hospital, AlAhsa, Saudi Arabia, from 2017 to 2021. The study included children (less than 14 years old) who presented to the emergency department with a history of foreign body ingestion. The biographical data, clinical presentation, type of foreign body, and X-ray findings were documented.

Results

A total of 91 cases of foreign body ingestion or aspiration in children under 14 years of age were included. Approximately half of the patients were under the age of three, and 62.2% of them were male, while 37.8% were female. The clinical presentation revealed that only 24% were symptomatic. Coins were the most commonly ingested foreign bodies (28.9%), followed by metallic objects (20%), and batteries were the least frequently ingested foreign bodies, recorded in eight cases.

Conclusion

Early detection and treatment of foreign body ingestion is crucial to prevent consequences. In this study, the most frequent foreign bodies detected were coins among children up to three years old. Raising parents’ awareness about the prevention of foreign body ingestion is an important step toward reducing its incidence.

## Introduction

Foreign body (FB) ingestion occurs most frequently in the pediatric age group. In the United States, coins are the most often ingested FBs; nonetheless, the ingestion of fish bones and other food-related objects is also prevalent. Children with mental health issues, boys in adolescence, and children under the age of five were the groups with the highest incidence of FB ingestion. Furthermore, young children may ingest foreign objects that are provided to them by, for example, older brothers [1-3]. Radiopaque objects, such as coins, screws, batteries, or toy pieces, are often ingested by children. However, the literature also records incidents of appendicitis caused by FBs that were lodged in the cecum. The majority of complications are triggered by the impaction of FBs in the esophagus, primarily in the case of anatomical abnormalities or underlying disorders [4,5].

The majority of ingested FBs easily pass through the digestive system without any complications; however, endoscopic or surgical intervention has been documented in a few children. The ideal circumstances and/or timing for performing these operations on youngsters are still up for dispute. Fortunately, the great majority of esophageal FBs are naturally eliminated with minimal complications; nevertheless, certain FBs cannot easily pass via the pylorus, stomach, duodenum, ileocecal valve, Meckel's diverticulum, and/or anus [5]; therefore, 10% of ingested FBs may remain in the GI tract [6,7]. Even if just for a short time, ingesting disc batteries may cause considerable injury if they get lodged in the stomach, nose, ear, or another orifice. Larger lithium-based batteries pose the most danger, although all models carry some risk. Cases of suspected button/disc battery ingestion are treated as medical emergencies; a battery lodged in the esophagus should be removed as soon as possible [8,9].

Medical expenditures and prognosis are typically favorable in pediatric FB ingestion, with most patients passing the ingested items without intervention. Mortality and morbidity are minimal in cases where patients receive any intervention. Ingesting high-risk objects (button batteries, magnets) might cause complications and, in rare circumstances, death [10-13].

Ingestions of foreign bodies (FBs) represent a challenging clinical dilemma for emergency departments (EDs), and 20% of patients need an endoscopy. We intended to document our experiences with FB ingestion at the Maternity and Children Hospital, AlAhsa, Saudi Arabia, where an emergency endoscopic service is accessible, taking into account the patient's age, sex, kind of FB material, comorbidities, and intervention.

## Materials and methods

An observational retrospective study was conducted to estimate the incidence of FB ingestion over four years (2017-2021) at the Maternity and Children Hospital, AlAhsa. The data were collected by revising the medical records of patients presenting to the emergency department with a history of FB ingestion. Patients between 0 and 14 years of age were included. On the other hand, the study excluded cases where the FB ingestion was located outside of the gastrointestinal (GI) tract or spontaneous elimination of the FB occurred. The data collected included demographical data, clinical presentation, radiological findings, and types of the FBs after removal by endoscopy were recorded. The data were then reviewed, coded, and entered into the statistical program IBM SPSS version 22. (SPSS, Inc. Chicago, IL). Two-tailed tests were used for all statistical analyses. A P-value of less than 0.05 was considered statistically significant. All variables, including patients' age, gender, FB data, and x-ray results, were subjected to descriptive analysis based on frequency and percent distribution. Crosstabulation was utilized to examine the relationship between FB ingestion, patients' age and gender, and FB clinical data, as well as the clinical presentation by type of ingested FB among the research participants. Pearson's chi-square test and the exact probability test for tiny frequency distributions were used to test the relationships.

## Results

In total, 91 pediatric cases diagnosed with FB ingestion were included. A total of 48 patients (52.7%) were under three years of age, 29 (31.9%) were four to six years old, and 14 (15.4%) were over six years of age. Moreover, 56 (62.2%) were males and 34 (37.8%) were females (Table 1).

**Table 1:** Personal characteristics of pediatric age group up to 14 years of age diagnosed with foreign body ingestion

Personal data	N	%
Age in years		
6 months-3 years	48	52.7%
4-6 years	29	31.9%
> 6 years	14	15.4%
Gender		
Male	56	62.2%
Female	34	37.8%

As for clinical presentation, FB ingestion was symptomatic among 22 (24.2%) patients in form of respiratory (72%) and gastrointestinal (8%), both (20%) symptoms and asymptomatic among 69 (75.8%). The most reported types of FB ingestion were coins (28.9%), followed by metallic FB (20%), and 8 cases (8.9%) involving ingested batteries. However, the FBs were unidentified in 18 (20%) cases. Overall, a total of 57 (63.3%) cases involved radiopaque FBs identified by x-ray (Table 2).

**Table 2:** Clinical data, presentation, and type of ingested foreign body among study patients

Clinical data	N	%
Clinical presentation		
Symptomatic	22	24.2%
Asymptomatic	69	75.8%
X-ray findings		
Radiopaque	57	63.3%
Radiolucent	33	36.7%
FB type		
Coin	26	28.9%
Metallic	18	20.0%
Unidentified	18	20.0%
Others	13	14.4%
Battery	8	8.9%
Sharp	7	7.8%

The most reported FBs among the cases of the younger patients (up to three years of age) were unidentified (27.1%), while 22.9% involved metallic FBs. In comparison, coins were the reported FB in 30.8% of the patients who aged more than six years old, while 23.1% had unidentified FBs with reported statistical significance (P = 0.009). Additionally, 47.9% of the younger cases had radiopaque FBs in comparison to 61.5% of the older cases (P = 0.001) (Table 3).

**Table 3:** Relation between FB ingestion cases age and FB clinical data P: Pearson X2 test $: Exact probability test * P < 0.05 (significant)

FB data	Age in years						P-value
	6 months-3 years		4-6 years		> 6 years		
	N	%	N	%	N	%	
FB type							.009*^$^
Coin	6	12.5%	16	55.2%	4	30.8%	
Metallic	11	22.9%	6	20.7%	1	7.7%	
Battery	4	8.3%	2	6.9%	2	15.4%	
Sharp	3	6.3%	2	6.9%	2	15.4%	
Others	11	22.9%	1	3.4%	1	7.7%	
Unidentified	13	27.1%	2	6.9%	3	23.1%	
Clinical presentation							.451
Symptomatic	14	29.2%	6	20.7%	2	14.3%	
Asymptomatic	34	70.8%	23	79.3%	12	85.7%	
X-ray findings							.001*^$^
Radiopaque	23	47.9%	26	89.7%	8	61.5%	
Radiolucent	25	52.1%	3	10.3%	5	38.5%	

Coins were the most reported ingested FB among males and females with no statistically significant difference (28.6% and 27.3%, respectively). In total, 30.4% of male cases displayed symptoms versus 14.7% of females (P = 0.049). Overall, 66.1% of males ingested radiopaque FBs in comparison to 57.6% of females (P = 0.423) (Table 4).

**Table 4:** Relation between FB ingestion cases gender and FB clinical data P: Pearson X2 test $: Exact probability test * P < 0.05 (significant)

FB data	Gender				P-value
	Male		Female		
	N	%	N	%	
FB type					.677^$^
Coin	16	28.6%	9	27.3%	
Metallic	10	17.9%	8	24.2%	
Battery	7	12.5%	1	3.0%	
Sharp	5	8.9%	2	6.1%	
Others	8	14.3%	5	15.2%	
Unidentified	10	17.9%	8	24.2%	
Clinical presentation					.049*
Symptomatic	17	30.4%	5	14.7%	
Asymptomatic	39	69.6%	29	85.3%	
X-ray findings					.423
Radiopaque	37	66.1%	19	57.6%	
Radiolucent	19	33.9%	14	42.4%	

The most reported FBs to show clinical presentation were the variant types (46.2%), followed by sharp FBs (28.6%), batteries (25%), and coins (23.1%) (Table 5).

**Table 5:** Clinical presentation by type of ingested FB among study cases P: Exact probability test

FB type	Clinical presentation				P-value
	Symptomatic		Asymptomatic		
	N	%	N	%	
Coin	6	23.1%	20	76.9%	.468
Metallic	3	16.7%	15	83.3%	
Battery	2	25.0%	6	75.0%	
Sharp	2	28.6%	5	71.4%	
Others	6	46.2%	7	53.8%	
Unidentified	3	16.7%	15	83.3%	

## Discussion

FB ingestion is very frequent in the pediatric age group, and the most reported emergencies are among children between six months and three years of age. Particularly, 80%-90% of FBs in the GI tract are asymptomatic and pass smoothly with no problems, 10%-20% are removed endoscopically, and 1% need surgical intervention due to complications [14]. Therefore, FB ingestion is a challenging clinical emergency in the pediatric age. The American Association of Poison Control Centers in 2002 reported that 75% of the >116,000 FB ingestions reported occurred in children aged ≤ 5 years [15].

The current study aimed to identify the incidence of FB ingestion among pediatric patients in the Maternity and Children Hospital in AlAhsa as it is a commonly encountered emergency worldwide. The study demonstrated that the incidence of FB ingestion was high among children of a younger age, while it was much lower among older children (above six years of age). Yalçin et al. [16] reported similar findings where the mean age was 2.27 ± 2.84 years old with a male: female ratio of 59/53. Moreover, Khorana et al. [17] found that 53.6% of their study cases were males while 46.4% were females with a median age of 43.5 months.

Regarding the type of ingested FBs, the current study ascertained that coins were the most reported FBs, followed by metallic FBs, while the incidence of battery ingestion was less than 10%. It is noteworthy that battery ingestion is the most dangerous and alarming case. Similar findings were reported by Cheng et al. [18] as the most commonly ingested FBs were coins (49%) and nonmetallic sharp objects (31%). Though x-rays could detect all metallic objects and 86% of glass objects, the sensitivity of fishbone detection was only 26%. Additionally, Dereci et al. [19] found that the most reported ingested FBs were coins, sewing pins, safety pins, and hair clips. The first pediatric cases reported in the literature included a nail clipper that was reported at the stomach and a sewing pin that penetrated through the duodenal wall and stuck to hepatic parenchyma. Pak et al. [20] found that the majority of the FBs were sharp bones and were situated in the oropharynx. In a study conducted in China with 1,265 children and in another study conducted in Belgium with 325 children, the rates of coin ingestion were found to be 47% and 27%, respectively [21,22]. Generally, the ingested FBs differ according to areas and countries, but coins, toys, magnets, and batteries are commonly the most reported [23,24].

Symptoms were more frequent among the younger cases (under three years of age), and this may be due to the prevalence of metallic FB ingestion in this age group. The symptoms may also be due to the ingestion of sharp objects, which causes injury or impaction of the ingested FB. The public site of impaction is in the upper esophagus at the cricopharyngeal muscle, which is recorded in 75% of all cases of FB impaction [25]. Moreover, objects may be impacted in the mid-esophagus at the level of the aortic arch or left main bronchus or in the lower esophagus at the gastro-esophageal junction [26]. In about 10% of cases, FBs may impact the intestines [27].

## Conclusions

Ingestion of FBs is a typical occurrence in the pediatric population presenting to the emergency department. Regardless of whether a patient is exhibiting symptoms or not, it poses both diagnostic and therapeutic obstacles. Overall, this study ascertained that FB ingestion in children seem to be more common at younger ages. Clinical signs are dependent on the type of FB swallowed, with the majority of youngsters being absolutely asymptomatic.
